# Development and comparison of multivariate diagnostic models for rapidly progressive central precocious puberty in girls: the role of serum osteocalcin

**DOI:** 10.3389/fendo.2025.1728132

**Published:** 2026-01-19

**Authors:** Wei Qin, Runqi Wang, Tao Xie, Yanfei Chen, Dan Zeng, Ziting Ding, Dan Lan

**Affiliations:** 1 Department of Pediatrics, The First Affiliated Hospital of Guangxi Medical University, Nanning, China; 2 Department of Pediatrics, The First People’s Hospital of Nanning, Nanning, China; 3 Difficult and Critical Illness Center, Pediatric Clinical Medical Research Center of Guangxi, Nanning, China; 4 The Key Laboratory of Children’s Disease Research in Guangxi’s Colleges and Universities, Education Department of Guangxi Zhuang Autonomous Region, Nanning, China

**Keywords:** diagnostic model, girls, nomogram, osteocalcin, rapidly progressive central precocious puberty

## Abstract

**Objectives:**

To develop a diagnostic prediction model for rapidly progressive central precocious puberty (RP-CPP) and evaluate the contribution of osteocalcin(OC) to the model.

**Methods:**

For a total of 411 girls who met the criteria for central precocious puberty were selected. Of these, 219 were included in the training set, 87 in the internal validation set, and 105 in the external validation set. Binary logistic regression was used to construct the model. The model fit and diagnostic accuracy were assessed using the Akaike Information Criterion (AIC), calibration curves, and the area under the receiver operating characteristic curve(AUC). The model was presented in the form of a nomogram. Internal and external validations of the model were performed.

**Results:**

Diagnostic models for RP-CPP were developed both with and without the inclusion of OC. Among all models, those that included OC consistently demonstrated smaller AIC values, higher AUC values, and lower prediction error rates. A model incorporating the duration of breast development, serum OC levels, mean ovarian volume, endometrial presence/absence, and breast Tanner staging demonstrated superior performance. The AUC for diagnosing RP-CPP was 0.973, with a sensitivity of 91.6% and specificity of 92.5%. The model performed well in the internal and external validation sets, demonstrating good clinical application value.

**Conclusion:**

The inclusion of OC helps improve the predictive performance of the model. For the diagnosis of RP-CPP in girls, a model can be chosen that includes the duration of breast development, serum OC levels, mean ovarian volume, endometrial presence/absence, and breast Tanner staging. However, all samples were from a single center, and multicenter validation is still needed.

## Introduction

Among patients with central precocious puberty (CPP), there are some special types, one of which is rapidly progressive central precocious puberty (RP-CPP). Epidemiological studies indicate that as the incidence of CPP increases, the prevalence of RP-CPP also shows a rising trend (1–4). The prominent clinical features of RP-CPP include advanced bone age (BA) and gonadal development, which may significantly impact adult height or psychological health. Early identification and proactive treatment of RP-CPP can bring greater benefits to patients, making early recognition particularly important. Therefore, the development of precise, rapid, and effective diagnostic methods remains an urgent priority for clinical researchers.

Various hormones and factors within the body interact to modulate the development of puberty. Researchers are investigating factors influencing pubertal progression and actively seeking biomarkers for early detection of RP-CPP. Zung et al. suggest that the measurement of morning urine LH levels is a non-invasive and reliable method. Using a cut-off value of 1.16 IU/L for morning urinary luteinizing hormone(LH) to identify RP-CPP, the sensitivity and specificity are 83% and 72%, respectively, which assists in distinguishing between slowly progressive CPP(SP-CPP) and RP-CPP (5). The study by Calcaterra et al. demonstrated that an ultrasonic breast volume of ≥0.85 cm^3^ is an independent predictor of RP-CPP (6). According to Chen Yun et al., girls with RP-CPP have more advanced BA, higher basal LH levels, and larger ovarian and uterine volumes compared to those with SP-CPP, with BA being the most helpful factor in identifying RP-CPP in girls (7). Zhang et al. ‘s research shows that when the basal LH is ≥0.58 IU/L, the sensitivity for diagnosing RP-CPP is 77.5% and the specificity is 66.7% (8). Research by Kim et al. also confirmed that advanced BA is a risk factor for pubertal progression in girls (9). Furthermore, quantitative pituitary stalk perfusion by arterial spin labeling is used as a non-invasive method to identify progressive CPP with a sensitivity of 76%, specificity of 83%, and accuracy of 77% (10).However, the existing research findings still fall short in terms of sensitivity and specificity for diagnosing RP-CPP. Given the suboptimal performance of single factors in identifying RP-CPP, a few studies have explored multi-factorial approaches for diagnosis. A model incorporating uterine volume, estradiol levels, endometrial thickness, BA, and breast volume measured by ultrasound demonstrated a sensitivity and specificity of 77.1% and 83.3%, respectively, for distinguishing RP-CPP (6). Chen et al. ‘s research has shown that the combination of anti-Müllerian hormone (AMH) and inhibin B can differentiate between slowly progressive CPP and progressive CPP with a sensitivity of 80% and a specificity of 89.3% (11). Despite these efforts, accurately identifying RP-CPP in its early stages remains a significant challenge.

Osteocalcin (OC) serves as a marker of bone turnover, participating in the regulation of osteoblast and osteoclast activity, and is associated with bone growth and mineralization. Moreover, the metabolically active form of OC functions as an endocrine hormone and may be involved in sexual development (12, 13). Our previous study has shown that in girls with CPP, changes in serum OC levels precede those of estradiol and BA, potentially serving as an early biomarker for RP-CPP. When using a cutoff value of 107.05 ng/mL for serum OC, the sensitivity and specificity for diagnosing RP-CPP were 91.1% and 70.7%, respectively (14).

Based on our previous research (14, 15), this study aims to develop a diagnostic prediction model for RP-CPP based on serum OC. We will develop separate models with and without OC to assess its impact on diagnostic performance and use multiple statistical approaches to select the optimal model. Additionally, a nomogram-based scoring system is constructed. This approach is designed to assist in developing a precise and efficient clinical diagnostic workflow.

### Study population

Girls diagnosed with CPP at the First Affiliated Hospital of Guangxi Medical University between January 2020 and December 2022 were included in the dataset for model construction. This dataset was randomly divided into a training set and a test set (internal validation set) at a ratio of 7:3. Girls diagnosed with CPP between January 2023 and October 2023 were included as the external validation set.

Diagnostic Criteria for CPP in Girls: 1. Onset of breast development before age 8 or menarche before age 10. 2. Advanced bone age, exceeding chronological age by ≥1 year. 3. Gonadal enlargement, as evidenced by ultrasound findings of enlarged uterus and ovaries. 4. Gonadotropin releasing hormone (GnRH) stimulation test indicating activation of the hypothalamic-pituitary-gonadal axis (LH ≥ 5 IU/L and LH/FSH ratio ≥ 0.6) (16, 17). Exclusion Criteria: 1. Incomplete examination data. 2. Negative GnRH stimulation test. 3. Prior exposure to GnRH agonist (GnRHa) treatment. 4. Secondary CPP (e.g., due to tumors, congenital adrenal hyperplasia [CAH]). 5. Presence of underlying diseases or a family history of abnormalities.

Diagnostic Criteria for RP-CPP: Progression from one Tanner stage to the next in less than 6 months, accompanied by advanced BA (BA exceeding chronological age by more than 1 year or a significant advancement in BA over a short period) (5, 18). Alternatively, the interval from breast development to menarche is less than 2 years (16).

Grouping: Among the girls diagnosed with CPP included in this study, those meeting the diagnostic criteria for RP-CPP were classified into the RP-CPP group, and the remainder were assigned to the non–rapidly progressive CPP (NRP-CPP) group.

## Methods

Data Extraction: Medical history records were obtained from the electronic medical record system to collect clinical information for girls diagnosed with CPP. The collected data included age, duration of breast development (time from breast development to initial consultation), height, weight, body mass index (BMI), birth weight, breast Tanner staging, and parental heights. Laboratory tests and imaging examinations.

BA assessment: BA was independently assessed by two experienced pediatric endocrinologists using the Greulich-Pyle (G.P.) atlas. The final BA was determined as the average of the two assessments. Ultrasonography Measurements: All girls underwent transabdominal ultrasonography to measure uterine length, endometrial thickness, and ovarian dimensions. Ovarian volume was calculated using the formula: Ovarian Volume (cm^3^) =0.5233×Length×Width×Depth (cm).

Variable Selection for the Model: Potential factors influencing precocious puberty in girls were identified through a comprehensive literature review, clinical experience, and statistical analysis. Clinical data were extracted and compiled into a dataset. Initial screening of these factors was performed using univariate analysis, with a significance level of P < 0.05 as the threshold. Following this, multivariate analysis was conducted to further refine the selection of variables for inclusion in the model. Various multivariate analysis methods were employed, including cross-validated LASSO regression, stepwise regression, Bayesian best subset selection, and random forest.

Model Construction: A binary logistic regression model was constructed to diagnose RP-CPP. The dependent variable was whether the patient was diagnosed with RP-CPP, and the independent variables were selected based on the optimal results from various multivariate screening methods. Our previous study showed that OC aids in the early identification of RP-CPP (14); therefore, we developed separate models with and without OC to assess its impact on diagnostic performance.

Sample Size Estimation for the Model: Based on previous analyses of risk factors associated with CPP, studies have shown that typically 3–6 risk factors are independently related to the occurrence of CPP (19). According to the empirical rule of 10 events per variable (10 EPV) (20), it is estimated that the risk prediction model developed in this study will include no more than 6 predictors. Therefore, a minimum of 60 positive cases (10 EPV × 6 predictors) is required to meet the sample size needs for model construction.

### Statistical analysis

Means between groups were compared using independent sample t-tests or analysis of variance (ANOVA). Proportions were compared using chi-square tests. For non-normally distributed data, Wilcoxon rank-sum tests were employed, statistical analyses were conducted using SPSS version 23.0, and a significance level of P < 0.05 was used for the initial screening of independent factors associated with RP-CPP. Multivariate analysis was conducted using cross-validated LASSO regression, stepwise regression, Bayesian best subset selection, and random forest methods. The results from these different screening methods were compared, and the optimal screening result was selected based on the AIC values. The final independent variables included in the model were determined based on this optimal result. A binary logistic regression model was constructed to estimate the regression coefficients for each predictor. The effect sizes of the regression model were expressed as odds ratios (ORs) with their corresponding 95% confidence intervals (CIs). The goodness-of-fit of the model was assessed using the Hosmer-Lemeshow test, P > 0.05 indicates adequate model fit. ROC curves were plotted and the AUC was used to evaluate the diagnostic performance of the model. Calibration plots were used to assess the consistency of the model’s predictive performance. The clinical utility of the model was evaluated using DCA. The model was presented in the form of a nomogram. Analyses were performed using R version 4.0.0. All tests were 2 sided, and P < 0.05 was considered to indicate statistical significance.

## Results

### Clinical characteristics

From a total of 1046 girls with precocious puberty, 411 girls who met the criteria for CPP were selected. Of these, a total of 306 girls with CPP (77 with RP-CPP and 229 with NRP-CPP) were included in the modeling dataset and were randomly divided into a training set (train) and an internal validation set (test) in a ratio of 7:3(219 were included in the training set, 87 in the internal validation set). An additional 105 girls with CPP (38 with RP-CPP and 67 with NRP-CPP) from a different time period were included as the external validation set (external). Comparison of clinical characteristics is detailed in **Table 1**.

**Table 1:** Comparison of clinical characteristics between the RP-CPP and NRP-CPP groups in the training set, test set, and external validation set.

Parameters	Training set			Test set			External validation set		
	RP-CPP (n=53)	NRP-CPP (n=166)	P	RP-CPP (n=24)	NRP-CPP (n=63)	P	RP-CPP (n=38)	NRP-CPP (n=67)	P
age (y)	8.80 ± 0.73	8.52 ± 0.94	0.046*	8.86 ± 0.61	8.43 ± 1.04	0.063	8.96±0.94	8.02±1.21	<0.001*
height (cm)	139.15 ± 5.46	132.75 ± 7.45	<0.001*	140.68 ± 7.12	132.58 ± 7.93	<0.001*	138.72±7.46	132.30±8.49	<0.001*
weight (kg)	35.50 ± 6.32	29.49 ± 5.93	<0.001*	35.41 ± 6.78	29.42 ± 5.00	<0.001*	35.21±6.40	29.89±6.60	<0.001*
duration of breast development (m)	9.36 ± 5.72	10.41 ± 8.23	0.388	9.71 ± 5.20	9.35 ± 6.98	0.817	9.82±4.64	11.45±8.38	0.202
BMI(kg/m2)	18.23 ± 2.44	16.61 ± 2.18	<0.001*	17.76 ± 2.38	16.64 ± 1.82	0.021*	18.15±1.99	16.90±2.14	0.004*
BA(y)	10.74 ± 0.91	9.78 ± 1.10	<0.001*	11.02 ± 1.00	9.46 ± 1.31	<0.001*	11.30±1.04	9.96±1.66	<0.001*
Δ(BA-CA)(y)	1.94 ± 0.78	1.26 ± 0.84	<0.001*	2.16 ± 0.78	1.03 ± 0.87	<0.001*	2.35±0.89	1.88±1.05	0.025*
IGF-1(ng/ml)	400.53±103.16	304.66±93.15	<0.001*	389.01±81.50	314.55±98.26	0.001*	281.61±78.37	201.45±66.76	<0.001*
OC(ng/ml)	140.81±36.56	87.13±25.71	<0.001*	143.75±34.13	78.93±25.65	<0.001*	136.90±49.93	98.93±31.60	<0.001*
Insulin (pmol/L)	113.92±51.42	83.61±40.46	<0.001*	104.42±53.23	89.23±67.91	0.327	133.09±90.02	109.94±99.75	0.300
TC(mmol/L)	4.04±0.64	4.24±0.60	0.033*	3.91±0.42	4.15±0.57	0.065	3.97±0.68	4.23±0.69	0.099
TG(mmol/L)	0.93±0.39	0.86±0.50	0.387	1.14±0.64	0.91±0.44	0.081	1.03±0.60	0.89±0.35	0.204
HDL (mmol/L)	1.27±0.25	1.39±0.30	0.008*	1.23±0.29	1.31±0.22	0.143	1.27±0.23	1.41±0.32	0.028*
LDL (mmol/L)	2.22±0.71	2.34±0.59	0.318	2.11±0.38	2.26±0.50	0.211	2.09±0.59	2.33±0.55	0.069
TSH(mIU/L)	2.35±1.24	2.37±1.20	0.908	2.13±1.11	2.34±0.95	0.403	2.46±1.46	2.47±0.97	0.956
FT4(pmol/L)	10.58±1.32	11.37±2.01	0.008*	10.74±1.61	11.40±1.89	0.132	14.80±2.30	15.35±2.45	0.298
FT3(pmol/L)	5.49±0.46	5.47±0.48	0.797	5.46±0.36	5.43±0.51	0.084	5.69±0.80	5.71±0.64	0.921
E2(pg/ml)	58.57±36.56	40.77±20.59	<0.001*	49.81±19.15	41.86±19.10	0.086	66.40±24.91	47.71±19.37	<0.001*
T(ng/ml)	0.28±0.18	0.20±0.12	0.001*	0.27±0.20	0.20±0.15	0.113	0.05±0.04	0.05±0.03	0.660
B-LH(mIU/ml)	5.21±6.43	1.65±2.00	<0.001*	4.97±4.11	1.65±1.72	<0.001*	4.49±3.35	2.09±3.01	<0.001*
B-FSH(mIU/ml)	5.60±2.72	4.12±2.08	<0.001*	6.05±2.71	4.20±2.26	0.002*	6.25±2.15	4.57±2.60	0.001*
uterine length (cm)	3.01±0.69	2.23±0.46	<0.001*	3.08±0.54	2.23±0.41	<0.001*	2.97±0.61	2.28±0.57	<0.001*
mean ovarian volume (cm3)	4.16±2.02	2.85±1.60	<0.001*	3.96±2.05	2.97±1.43	0.013*	4.02±2.32	2.87±1.75	0.005*
Breast Tanner stage									
Tanner stage 2	15(28.3%)	117(70.5%)	<0.001*	7(29.2%)	46(73.0%)	<0.001*	8(21.1%)	45(67.2%)	<0.001*
> Tanner stage 2	38(71.7%)	49(29.5%)		17(70.8%)	17(27.0%)		30(78.9%)	22(32.8%)	
endometrium									
Yes	38(71.7%)	14(8.4%)	<0.001*	15(62.5%)	3(4.8%)	<0.001*	29(76.3%)	20(29.9%)	<0.001*
No	15(28.3%)	152(91.6%)		9(37.5%)	60(95.2%)		9(23.7%)	47(70.1%)	

RP-CPP, rapid progressive central precocious puberty; NRP-CPP, Non-rapid progressive central precocious puberty; P, P value, for the comparison between RP-CPP and NRP-CPP; *P value <0.05; Duration of breast development, from the onset of breast development to first visit; BA, bone age; CA, chronological age; Δ(BA-CA), Δ (bone age − chronological age); IGF-1, insulin-like growth factor-1; OC, osteocalcin; TC, total cholesterol; TG, Triglyceride; HDL, high-density lipoprotein; LDL, Low-density lipoprotein; TSH, thyroid-stimulating hormone; FT4, free thyroxine; FT3, free triiodothyronine; E2, estradiol; T, testosterone;B-LH, basal luteinizing hormone; B-FSH, basal follicle-stimulating hormone.

In the modeling dataset, the mean age of the RP-CPP group was slightly higher than that of the NRP-CPP group (8.87 ± 0.73 vs. 8.29 ± 0.94 years, P < 0.001). Although the duration of breast development was shorter in the RP-CPP group (9.47 ± 5.53 vs. 11.18 ± 8.38 months, P = 0.042), the difference between bone age and chronological age(Δ (BA-CA)) was significantly greater in the RP-CPP group (1.95 ± 0.80 vs. 1.34 ± 0.86 years). The distribution of breast Tanner stages was as follows: in the RP-CPP group, Tanner stage 2 accounted for 28.57%, Tanner stage 3 for 53.25%, and Tanner stage 4 for 18.18%; in the NRP-CPP group, Tanner stage 2 accounted for 71.18%, Tanner stage 3 for 26.14%, and Tanner stage 4 for 2.68%. The RP-CPP group was predominantly in Tanner stage 3, whereas the NRP-CPP group was predominantly in Tanner stage 2. The relevant laboratory indicator results are shown in **Table 1**.

Based on the results of univariate statistical analysis, the following potential predictive factors were initially screened: age, duration of breast development, BA, Δ(BA-CA), height, weight, body mass index (BMI), Breast development at Tanner stage 2 (yes/no), insulin-like growth factor 1 (IGF-1), OC, fasting insulin, total cholesterol(TC), high-density lipoprotein (HDL), free thyroxine (FT4), estradiol (E2), testosterone(T), basal luteinizing hormone (B-LH), basal follicle-stimulating hormone (B-FSH), uterine length, mean ovarian volume, and the presence or absence of endometrium.

### Multivariate variable selection and model construction

Multivariate variable selection was performed using cross-validated LASSO regression, stepwise regression, Bayesian best subset selection, and random forest methods. Additionally, diagnostic models with and without OC were developed (**Table 2**).

**Table 2:** Results of model selection using cross-validated LASSO regression, stepwise regression, bayesian best subset selection, and random forest methods in the training set.

Parameter	Method	Selection results	
Include OC	CV-LASSO	Model	duration of development+OC+mean ovarian volume+ En+Tanner Stage 2 (P = 0.634; AIC = 83.32; Error rate:Train=5.48%, Test=8.05%)
		AUC(95%CI)	Train:0.973(0.950-0.996);Test:0.972(0.939-1.000);External:0.923(0.869-0.972)
	Stepwise	Model	duration of development+OC+ En+Tanner Stage 2 (P = 0.965; AIC = 90.55; Error rate:Train=6.39%, Test=6.90%)
		AUC(95%CI)	Train:0.966(0.944-0.989);Test:0.967(0.923-1.000);External:0.906(0.851-0.962)
	Best Subset	Model	duration of development+BA+OC+HDL+mean ovarian volume+En+Tanner Stage 2 (P = 0.695; AIC = 76.55; Error rate:Train=5.02%, Test=5.75%)
		AUC(95%CI)	Train:0.981(0.961-1.000);Test:0.972(0.934-1.000);External:0.930(0.884-0.976)
	Random Forest	Model	OC+En (P = 0.526; AIC = 111.24; Error rate:Train=8.68%, Test=5.75%)
		AUC(95%CI)	Train:0.942(0.903-0.981);Test:0.981(0.952-1.000);External:0.825(0.747-0.903)
Exclude OC	CV-LASSO	Model	duration of development+mean ovarian volume+En+ Tanner Stage 2 (P = 0.248; AIC = 108.89; Error rate: Train=12.33%, Test=14.94%)
		AUC(95%CI)	Train:0.949(0.914-0.985);Test:0.868(0.769-0.968);External:0.874(0.808-0.939)
	Stepwise	Model	duration of development+mean ovarian volume+ En+Tanner Stage 2 (P = 0.055; AIC = 99.09; Error rate: Train=10.05%, Test=12.64%)
		AUC(95%CI)	Train:0.963(0.934-0.991);Test:0.895(0.810-0.980);External:0.901(0.845-0.958)
	Best Subset	Model	duration of development+BA+mean ovarian volume+En+Tanner Stage 2 (P = 0.439; AIC = 103.88; Error rate: Train=9.59%, Test=13.79%)
		AUC(95%CI)	Train:0.956(0.925-0.988);Test:0.899(0.812-0.985);External:0.886(0.825-0.947)
	Random Forest	Model	duration of development+HDL+ mean ovarian volume+En+Tanner Stage 2 (P = 0.270; AIC = 104.13; Error rate: Train=11.42%, Test=14.94%)
		AUC(95%CI)	Train:0.956(0.923-0.990);Test:0.876(0.781-0.970);External:0.895(0.836-0.954)
Exclude duration of breast development	CV-LASSO	Model	OC +uterine length+En (P = 0.856; AIC = 110.06; Error rate: Train=8.68%, Test=2.30%)
		AUC(95%CI)	Train:0.946(0.910-0.982);Test:0.985(0.958-1.000);External:0.841(0.768-0.915)
	Stepwise	Model	OC+mean ovarian volume +En (P = 0.475; AIC = 108.53; Error rate: Train=7.31%, Test=6.90%)
		AUC(95%CI)	Train:0.947(0.911-0.984);Test:0.989(0.970-1.000);External:0.830(0.754-0.906)
	Best Subset	Model	OC+mean ovarian volume +En (P = 0.475; AIC = 108.53; Error rate: Train=7.31%, Test=6.90%)
		AUC(95%CI)	Train:0.947(0.911-0.984);Test:0.989(0.970-1.000);External:0.830(0.754-0.906)
	Random Forest	Model	OC+En (P = 0.526; AIC = 111.24; Error rate:Train=8.68%, Test=5.75%)
		AUC(95%CI)	Train:0.942(0.903-0.981);Test:0.981(0.952-1.000);External:0.825(0.747-0.903)

CV-LASSO, cross-validated LASSO regression; Stepwise, Stepwise regression; Best Subset, Bayesian Best Subset Selection; AIC, Akaike Information Criterion; duration of development, duration of breast development; Tanner Stage 2, breast development of Tanner stage 2; BA, bone age; OC, osteocalcin; HDL, high-density lipoprotein; En, endometrium; P, Hosmer and Lemeshow goodness of fit (GOF) test result for the model; Train, training set; Test, internal validation set; External, external validation set; Error rate(train), prediction error rate of the training set; Error rate(test), prediction error rate of internal validation set; AUC(95%CI), Area under the ROC curve and its 95% confidence interval.

**Figure 1:**
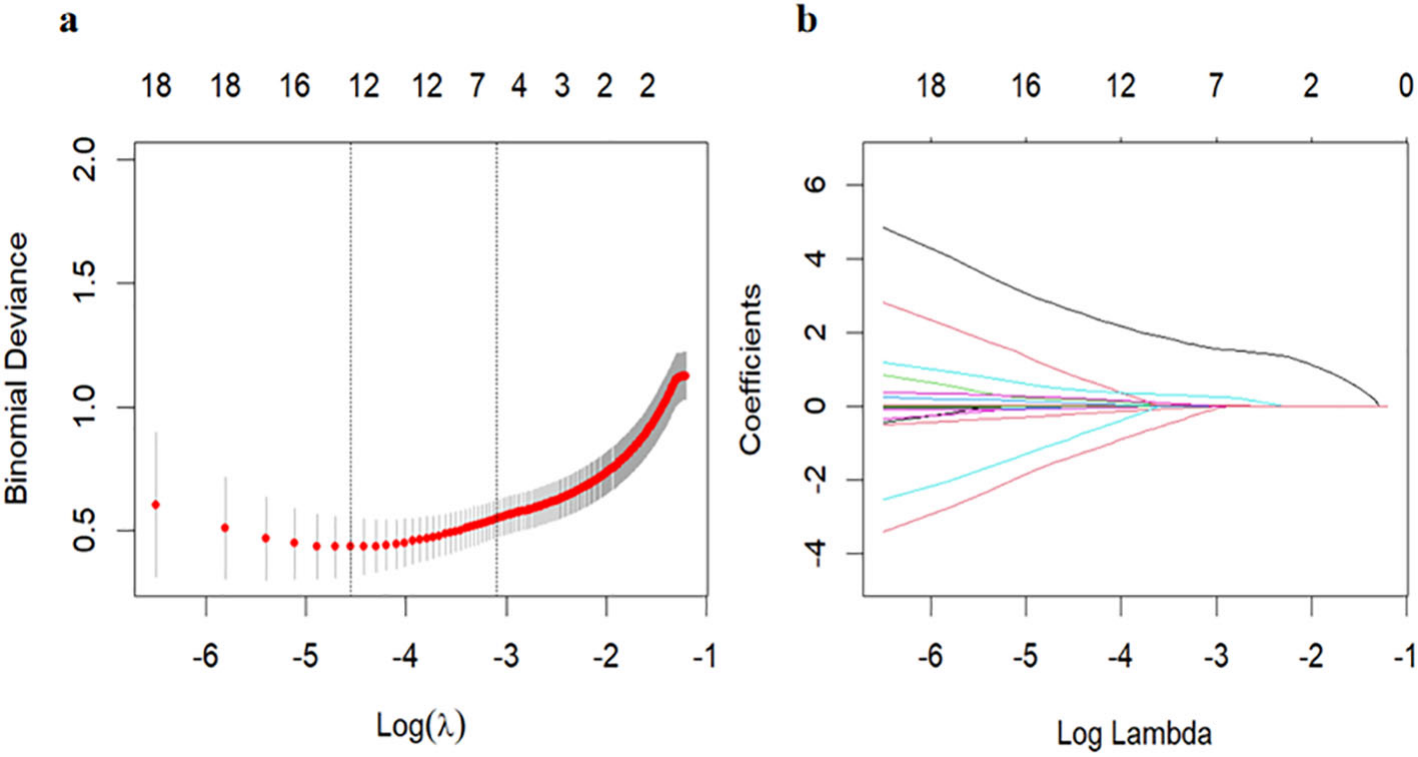
Mean square error of the model under different lambda, Loglambda and correlation coefficient. **(a)** Mean squared error (MSE) of the model across different λ values. The x-axis shows log(λ), and the y-axis shows MSE. Three-fold cross-validation was used for parameter selection in LASSO regression, with plots of partial likelihood deviance (binomial deviance) and log(λ). The left vertical dashed line indicates the log(λ) value corresponding to the minimum MSE; the right dashed line marks the largest λ within one standard error of the minimum MSE. Using the optimal λ, five predictors with non-zero coefficients were selected. **(b)** Log(λ) versus regression coefficients. LASSO regression was applied for variable selection among 21 candidate predictors. The plot displays the coefficient paths of all variables as a function of log(λ).

In the training set, when OC was included, the screening results indicated that the Bayesian best subset selection method had the smallest AIC value (AIC = 76.55), suggesting a relatively optimal model, followed by cross-validated LASSO regression (AIC = 83.32). The screening results are shown in in **Table 2** and **Figure 1**. Both models exhibited high AUC values and low prediction error rates in the training set, internal validation set, and external validation set. Given that the model selected by cross-validated LASSO regression included 5 variables, while the model selected by Bayesian best subset selection included 7 variables, the model selected by cross-validated LASSO regression is more convenient for clinical application. When OC was excluded from the dataset, the stepwise regression method yielded the best screening results, but the AIC value increased to 99.09, and the number of included variables increased to 6. The AUC in the training set was 0.963(the sensitivity and specificity were 93.4% and 90.6%, respectively), but the AUC in the internal validation set decreased to 0.895, and the prediction error rates in both the training set and validation set increased. Therefore, the inclusion of OC contributes more effectively to enhancing the diagnostic performance of the model. The model selected by cross-validated LASSO regression was identified as the optimal model (Model-1), comprising: duration of breast development, serum OC levels, mean ovarian volume, endometrial presence/absence, and breast Tanner staging (whether the breast development is at Tanner Stage 2 or not) (**Tables 2**, **3**).

**Table 3:** Correlation coefficients, odds ratios (ORs) and 95% confidence intervals for the two optimal models.

Models	Independent variables	R	OR (95%CI)	P	AIC
Model-1	(Include OC)				83.32
	(Intercept)	-5.60			
	duration of development	-0.32	0.73 (0.61∼0.83)	<0.001	
	OC	0.05	1.05 (1.03∼1.08)	<0.001	
	mean ovarian volume	0.54	1.71 (1.20∼2.58)	0.006	
	En	4.86	128.43 (22.02∼1217.04)	<0.001	
	Tanner Stage 2	-3.20	0.04 (0.005∼0.21)	<0.001	
Model-2	(Exclude duration of breast development)				108.53
	(Intercept)	-9.75			
	OC	0.06	1.06 (1.04∼1.09)	<0.001	
	mean ovarian volume	0.31	1.37 (1.03∼1.83)	0.031	
	En	2.51	12.34 (4.40∼37.91)	<0.001	

AIC, Akaike information Criterion; r, Correlation Coefficients; duration of development, duration of breast development; Tanner Stage 2, breast development of Tanner stage 2; BA, bone age; OC, osteocalcin; En, endometrium.

Given that Model 1 included duration of breast development—a variable highly susceptible to subjective bias and difficult to measure accurately—we excluded it from the dataset and rebuilt the model. In the training set, the screening results indicated that the stepwise regression and the Bayesian best subset selection method had the same results and the smallest AIC value (AIC = 108.53), suggesting a relatively optimal model. The optimal model (Model-2) was included the following variables: serum OC levels, mean ovarian volume, endometrial presence/absence (**Tables 2**, **3**).

### Evaluation of model diagnostic performance

**Figure 2:**
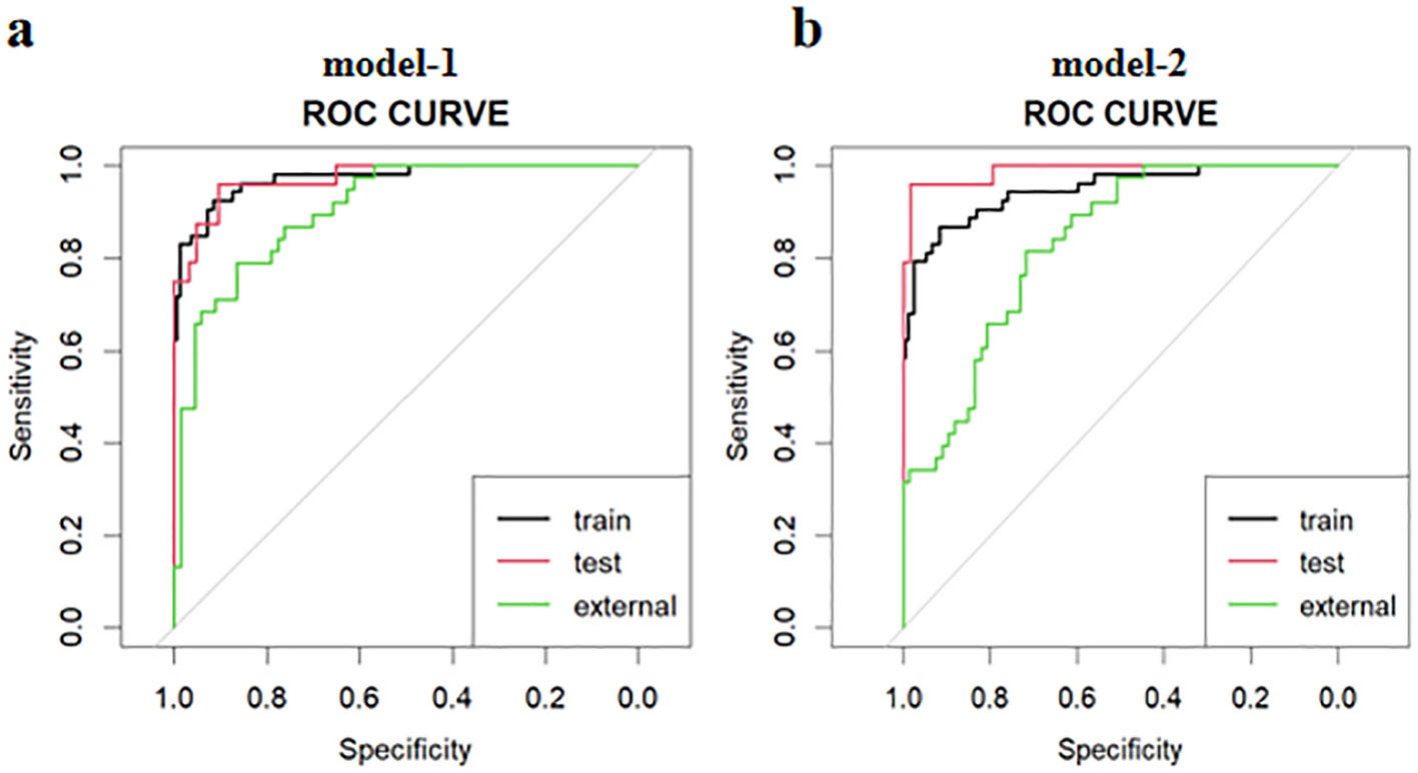
The ROC curves for diagnosing RP-CPP of model-1 and model-2 in the training set, test set, and external validation set. **(a)** The AUC of Model-1 in the training set, test set, and external validation set were 0.973(95%CI:0.950-0.996), 0.972(95%CI:0.939-1.000), and 0.923(95%CI:0.869-0.972), respectively, indicating high diagnostic performance. **(b)** Model-2 demonstrated high diagnostic performance in both the training and test sets, with AUCs of 0.947 (95% CI: 0.911-0.984) and 0.989 (95% CI: 0.970-1.000), respectively; however, performance declined slightly in external validation (AUC: 0.830; 95% CI: 0.754-0.906).

**Figure 3:**
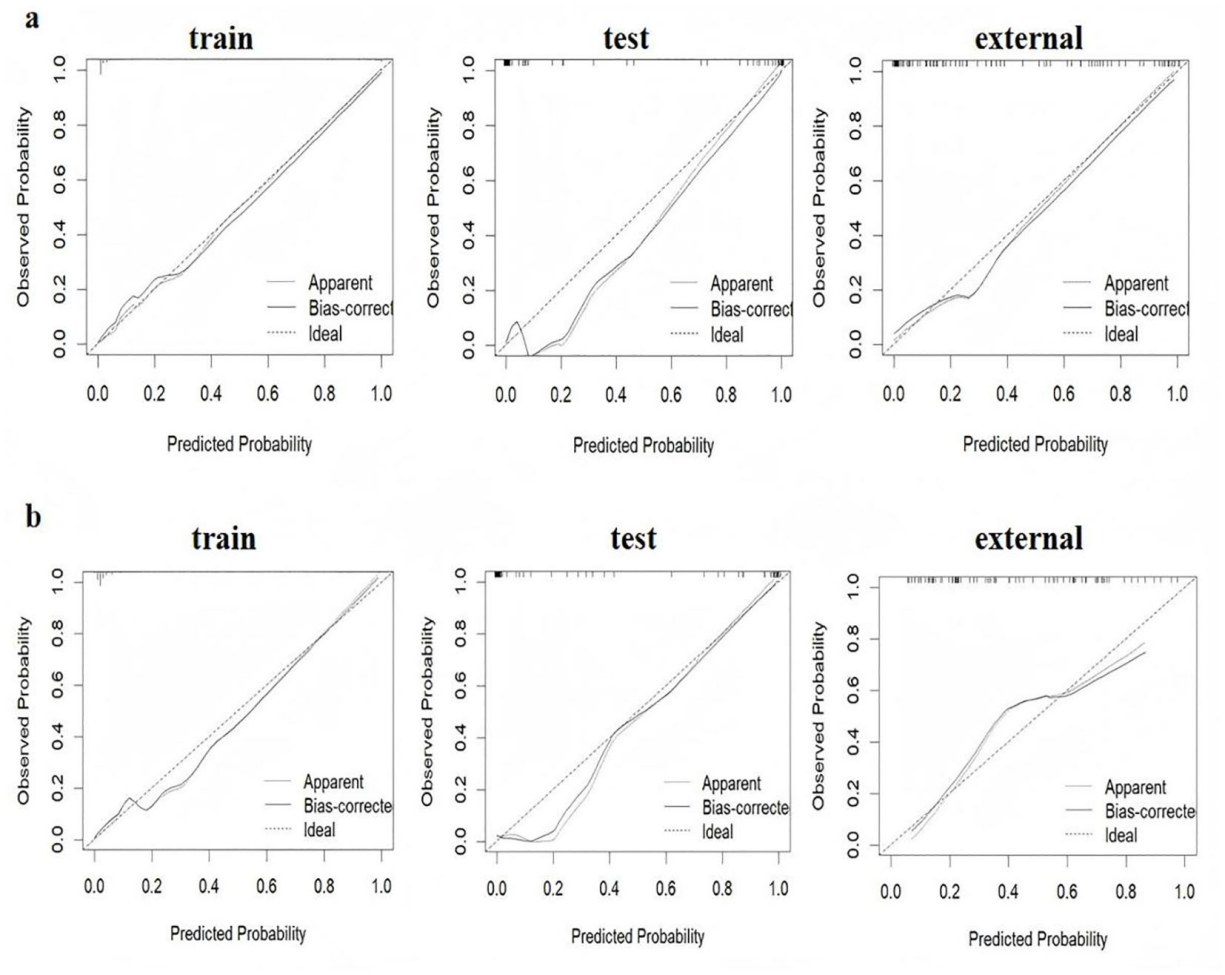
Calibration curves of model-1 and model-2 in the training set, test set, and external validation set. **(a)** Model-1 shows good consistency in predictive ability across the training set and external validation set, indicating good agreement between predicted and observed probabilities for RP-CPP. Although slight deviation was noted in the test set, overall calibration performance remained satisfactory. **(b)** The calibration curves for Model 2 in the training and test sets closely followed the ideal reference line, indicating good agreement between predicted and observed probabilities; however, some deviation was observed in the external validation set, suggesting only moderate calibration performance.

**Figure 4:**
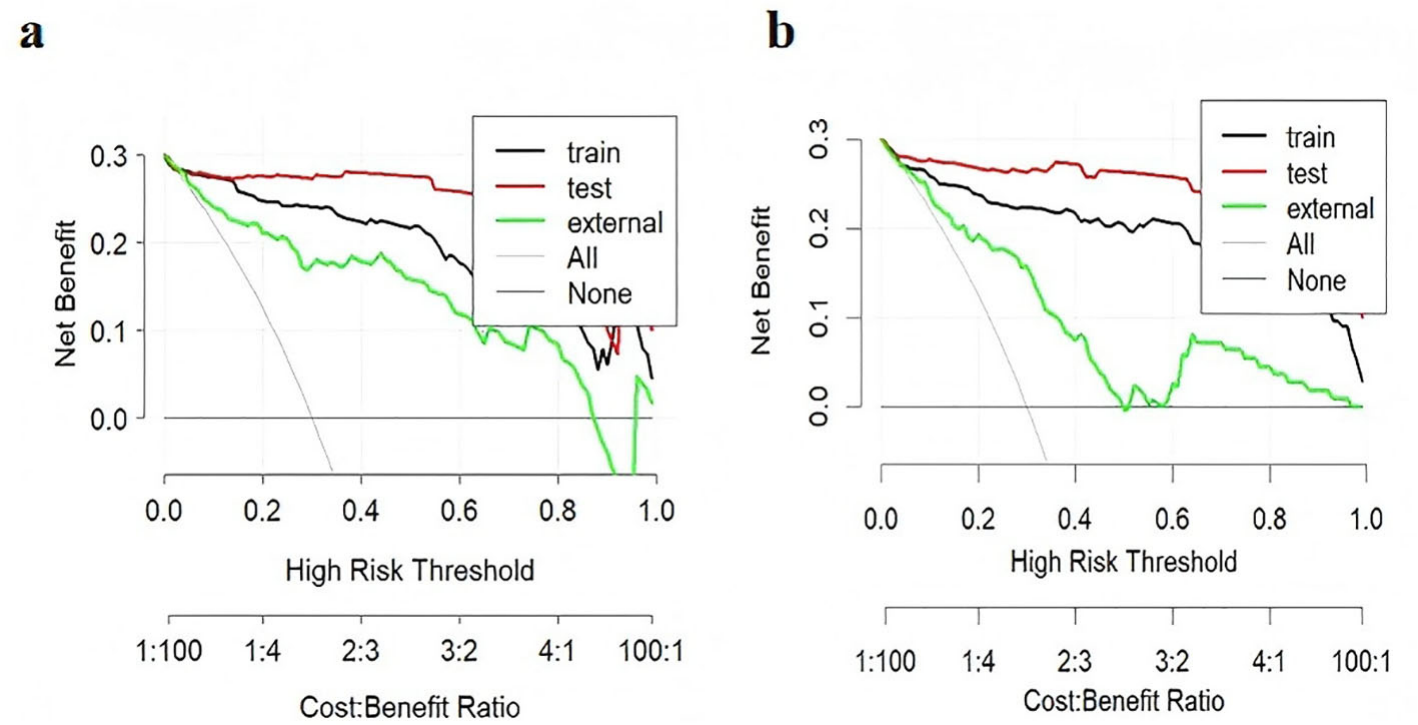
Clinical decision curve analysis (DCA) of the models. In the DCA, the y-axis represents net benefit (NB). The horizontal line indicates a net benefit of zero, corresponding to the strategy of treating no patients. The upward-sloping curve represents the net benefit when all patients are treated. A higher net benefit reflects greater clinical utility of the model. **(a)** DCA for Model-1; **(b)** DCA for Model-2. The DCA curves for both models lie above the reference lines, demonstrating good clinical decision-making performance in the training and test sets, though with slightly reduced performance in external validation.

The ROC curves for diagnosing RP-CPP using Model-1 and Model-2 are presented in **Figure 2**. The AUC of Model-1 in the training set, test set, and external validation set were 0.973(95%CI:0.950-0.996), 0.972(95%CI: 0.939-1.000), and 0.923(95%CI:0.869-0.972), respectively, indicating high diagnostic performance.Model-2 demonstrated high diagnostic performance in both the training and test sets, with AUCs of 0.947 (95% CI: 0.911-0.984) and 0.989 (95% CI: 0.970-1.000), respectively; however, performance declined slightly in external validation (AUC: 0.830; 95% CI: 0.754-0.906).The AUC, cut-off values, sensitivity, and specificity of the models are detailed in **Table 4**. The calibration plot for Model 1 is shown in **Figure 3a**. The curves for both the training and external validation sets closely followed the ideal reference line, indicating good agreement between predicted and observed probabilities for RP-CPP. Although slight deviation was noted in the internal validation set, overall calibration performance remained satisfactory. The calibration plot for Model 2 is shown in **Figure 3b**. Calibration curves for the training and test sets closely approximated the ideal reference line, indicating good agreement between predicted and observed probabilities. However, some deviation was observed in the external validation set, suggesting only moderate calibration performance. The decision curve analysis (DCA) is shown in **Figure 4**, both models’ DCA curves lie above the two reference lines, indicating favorable performance, particularly strong clinical utility in the training and test sets, though slightly reduced in external validation.

**Table 4:** AUC, cut-off values, sensitivity, and specificity of model-1 and model-2.

Models		AUC (95%CI)	Cut-off Values	Sensitivity	Specificity
Model-1	Train	0.973(0.950-0.996)	0.255	91.6%	92.5%
	Test	0.972(0.939-1.000)	0.092	90.5%	95.8%
	External	0.923(0.869-0.972)	0.691	86.4%	83.3%
Model-2	Train	0.947(0.911-0.984)	0.335	91.6%	86.8%
	Test	0.989(0.970-1.000)	0.368	98.4%	95.8%
	External	0.830(0.754-0.906)	0.244	71.6%	81.6%

Train, training set; Test, test set; External, external validation set.

### Nomogram

**Figure 5:**
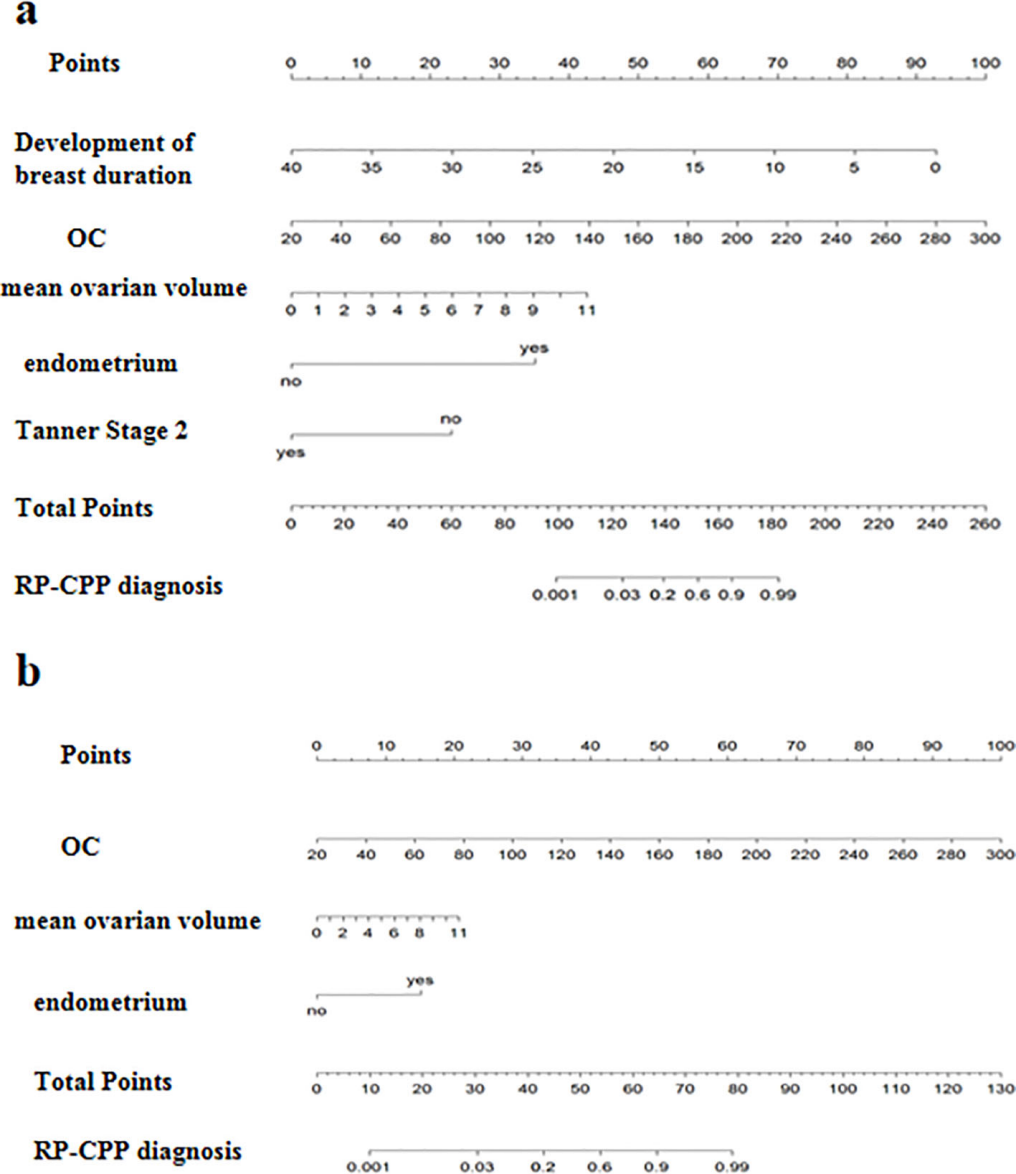
The nomograph of model-1 and model-2. **(a)** The nomograph of model-1; **(b)** The nomograph of model-2.

Nomograms for predicting RP-CPP using Model-1 and Model-2 are illustrated in **Figure 5**. The nomogram for Model-1 was developed based on five independent factors: duration of breast development, serum OC levels, mean ovarian volume, endometrial presence/absence, and whether at breast Tanner stage 2. The nomogram for Model-2 was developed based on three independent factors: serum OC levels, mean ovarian volume, endometrial presence/absence. In these nomograms, a higher total score, calculated as the sum of points assigned to each factor, is indicative of a greater probability of RP-CPP.

## Discussion

RP-CPP is characterized by the rapid progression of both growth and sexual development. Currently, clinical identification of RP-CPP relies mainly on the assessment of Tanner stages of sexual development and the rapid advancement of bone age. However, these methods generally require a period of observation to accurately evaluate the rate of development, and therefore, there remains a lack of effective early identification methods for RP-CPP. Several studies have attempted to evaluate RP-CPP using parameters such as bone age, basal LH levels, IGF-1, inhibin B (INHB), anti-Müllerian hormone(AMH), and the size of the uterus or ovaries (5–7, 11, 21, 22). However, the findings from these studies indicate that reliance on a single factor remains inadequate for the early identification of RP-CPP. Endocrine disorders affecting pubertal development in girls are influenced by a complex interplay of genetic, environmental, nutritional, and psychosocial factors (3, 23–26). Given the inherent limitations of single-predictor approaches, integrating multiple variables may enhance diagnostic accuracy. This notion is reinforced by a recent review highlighting the challenges in differentiating CPP from benign variants and proposing a structured diagnostic framework that combines clinical features, hormonal assessments, and imaging findings (27). Therefore, this study aimed to develop a diagnostic prediction model for RP-CPP to explore methods for early identification.

To date, a limited number of studies have explored the use of multivariate analysis methods for the early diagnosis of RP-CPP. For instance, Calcaterra et al. developed a model combining uterine volume, estradiol levels, uterine endometrium, BA, and breast volume measured by ultrasound. This model achieved a C-statistic of 0.86 for distinguishing RP-CPP, with a sensitivity of 77.1% and a specificity of 83.3% (6).Another study evaluated the diagnostic value of various parameters for RP-CPP and found that basal LH ≥ 0.2 IU/L, estradiol (E_2_) ≥ 50 pmol/L, uterine length ≥ 3.5 cm, uterine width ≥ 1.5 cm, presence of endometrial echoes, and ovarian volume ≥ 2 cm^3^ were significantly associated with RP-CPP. The study further showed that diagnostic performance improved with the inclusion of more combined parameters: when three or more were present, the AUC was 0.71 (sensitivity 58%, specificity 85%) (21).The study by Chen et al. indicated that girls with progressive CPP exhibit lower levels of AMH and higher levels of INHB compared to those with slowly progressive CPP. The combined use of AMH and INHB for distinguishing between slowly progressive CPP and progressive CPP yielded an AUC of 0.92, the sensitivity and specificity were 80% and 89.3%, respectively (11). Despite the efforts of researchers to investigate various methods, current approaches for identifying RP-CPP remain insufficient, exhibiting suboptimal sensitivity and specificity.

Our earlier study showed that serum OC levels change earlier than estradiol and BA, suggesting its potential as an early biomarker for RP-CPP (14). Therefore, this study constructs distinct models with and without OC to validate whether the inclusion of OC can enhance the diagnostic performance of the models. Our findings indicate that, when OC is included as a variable in the dataset, the optimal model (Model 1) achieves an AIC of 83.32, which is superior to the optimal model that excludes OC (selected through stepwise regression, AIC = 99.09) and involves a smaller number of independent variables. Additionally, Model 1 demonstrates a higher AUC in the internal validation set compared to the model excluding OC (0.972 vs. 0.895). Moreover, the model incorporating OC had significantly lower prediction error rates in both the training set and internal validation set compared to the model excluding OC (training set: 5.48% vs 10.05%; internal validation set: 8.05% vs 12.64%). These findings suggest that OC contributes to enhancing the predictive performance of the models and may be beneficial for the early identification of RP-CPP.

In this study, cross-validated LASSO regression, stepwise regression, Bayesian optimal subset selection, and random forest methods were employed to screen independent variables. When the dataset includes the OC, the model selected by Bayesian optimal subset exhibited the smallest AIC value (76.55), outperforming those identified by cross-validated LASSO regression (AIC = 83.32), stepwise regression (AIC = 90.55), and random forest (AIC = 111.24).However, the Bayesian best subset model included 7 predictors, compared with 5 in the cross-validated LASSO model. Their AUCs were 0.981 vs. 0.973 in the training set (P > 0.05), both 0.972 in internal validation, and 0.930 vs. 0.923 in external validation (P > 0.05).This indicates that both models have similar diagnostic efficacy. Considering the convenience of clinical application, a model that incorporates fewer independent variables is preferred. Therefore, the model selected by cross-validated LASSO regression (Model 1) is considered superior. It achieved an AUC of 0.973 in the training set, with 91.6% sensitivity and 92.5% specificity, and showed robust performance in both internal and external validation. Given the substantial subjectivity in assessing breast development duration, we also reconstructed models excluding this variable. The results indicate that the model (Model 2) incorporating OC, mean ovarian volume, and endometrial presence/absence was optimal, as it yielded the lowest AIC value(108.53). This model achieved an AUC of 0.947 in the training set, with a sensitivity of 91.6% and specificity of 86.8%. Our findings indicate that both Model 1 and Model 2 exhibit high AUC, sensitivity, and specificity in training and validation sets. Goodness-of-fit assessments confirm excellent model performance, calibration curves demonstrate strong predictive consistency, and decision curve analysis underscores their clinical utility. However, if OC measurement is unavailable, an alternative model selected via stepwise regression (AIC = 99.09) can be used. This model includes duration of breast development, BA, HDL, mean ovarian volume, endometrial presence/absence, and whether at breast Tanner stage 2. It achieved an AUC of 0.963 in the training set (sensitivity: 93.4%; specificity: 90.6%) and demonstrated good predictive performance in both internal (AUC: 0.895) and external validation (AUC: 0.901).In summary, Model 1 and Model 2 are complementary and address distinct clinical needs. We recommend that clinicians select the model best aligned with their institutional resources and data availability, favoring Model 1 when accurate measurement of breast development duration is feasible.

In conclusion, this study adopted multiple methods to screen independent variables and selected the optimal model. Both internal and external validation tests confirmed that the model demonstrates excellent fitting performance and significant clinical utility. A nomogram was developed as a scoring system to facilitate clinical application. Moreover, our preliminary research indicated that OC might be beneficial for the early identification of RP-CPP. By incorporating OC into the model, we confirmed its ability to enhance predictive performance. Therefore, the inclusion of OC in the model may be applicable for the early diagnosis of RP-CPP.

The limitation of this study is that the samples analyzed were all sourced from a single center. Whether the findings are generalizable to other centers remains to be verified through multicenter, large-sample clinical validations.

## Data Availability

The raw data supporting the conclusions of this article will be made available by the authors, without undue reservation.
